# Endocrine-disrupting chemicals and risk of diabetes: an evidence-based review

**DOI:** 10.1007/s00125-018-4621-3

**Published:** 2018-05-09

**Authors:** P. Monica Lind, Lars Lind

**Affiliations:** 1 Occupational and Environmental Medicine, Department of Medical Sciences, Uppsala University, Uppsala, Sweden; 2 Cardiovascular Epidemiology, Department of Medical Sciences, Entrance 40, Uppsala University, 751 85 Uppsala, Sweden

**Keywords:** Bisphenol A, BPA, Chemicals, DDE, DDT, Diabetes, EDCs, Endocrine-disrupting chemicals, Pesticides, Review

## Abstract

The purpose of this study was to review the epidemiological and experimental evidence linking background exposure to a selection of environmental endocrine-disrupting chemicals (EDCs) with diabetes and impaired glucose metabolism. The review summarises the literature on both cross-sectional and prospective studies in humans, as well as experimental in vivo and in vitro studies. The findings were subjected to evidence grading according to the Grading of Recommendations Assessment, Development and Evaluation (GRADE) classification. We found >40 cross-sectional and seven prospective studies regarding EDCs and risk of diabetes. Taken together, there is moderate evidence for a relationship between exposure to dichlorodiphenyldichloroethylene (*p*,*p*′-DDE), a metabolite of the pesticide dichlorodiphenyltrichloroethane, and diabetes development. Regarding polychlorinated biphenyls (PCBs), it is likely that the rodent models used are not appropriate, and therefore the evidence is poorer than for *p*,*p*′-DDE. For other EDCs, such as bisphenol A, phthalates and perfluorinated chemicals, the evidence is scarce, since very few prospective studies exist. Brominated flame retardants do not seem to be associated with a disturbed glucose tolerance. Thus, evidence is accumulating that EDCs might be involved in diabetes development. Best evidence exists for *p*,*p*′-DDE. For other chemicals, both prospective studies and supporting animal data are still lacking.

**Electronic supplementary material:**

The online version of this article (10.1007/s00125-018-4621-3) contains a slide of the figure for download, which is available to authorised users.

## Introduction

The prevalence of diabetes is increasing in all countries and the disease is becoming a substantial public health concern worldwide. During the last decade, numerous studies have proposed links between endocrine-disrupting chemicals (EDCs) and disturbances in glucose metabolism. The present review will focus on a representative selection of groups of environmental EDCs and use a well-known system to grade the evidence. We searched PubMed for publications with the search terms contaminant/pollutant/name of the chemicals included in the review [AND] diabetes/glucose/insulin, including both human and experimental studies.

## Overview of EDCs

An EDC has been defined as ‘an exogenous agent that interferes with synthesis, secretion, transport, metabolism, binding action or elimination of natural blood-borne hormones that are present in the body and are responsible for homeostasis, reproduction and developmental process’ [1]. EDC was originally a term devoted to disruption of the reproductive system but has now been broadened to include disturbances in other hormonal systems.

Some EDCs are lipophilic, such as polychlorinated biphenyls (PCBs), dioxins, organochlorine pesticides and brominated flame retardants (BFRs); they accumulate in the food chain and are ingested by humans. These chemicals are stored in adipose tissue and generally have very long half-lives (months to many years). Perfluoroalkyl and polyfluoroalkyl substances (PFASs) comprise another class of EDCs, the members of which are non-lipophilic and are transported bound to albumin and stored in the liver in humans. PFASs also accumulate in the food chain and, depending on the length of their carbon chain, also have very long half-lives (months to years) in humans. Together, these types of chemicals accumulating in humans are denoted persistent organic pollutants (POPs).

However, not all EDCs are persistent and not all accumulate to a major degree in humans. Bisphenol A (BPA), a well-known plastic hardener, has a very short half-life (hours) in humans but detectable levels are nevertheless found in most humans in the industrialised world due to exposure on a more-or-less daily basis. The phthalates are another huge group of EDCs with short half-lives (hours to days) but ubiquitous exposure. These chemicals are used as plasticisers and solvents.

The present review will focus on selected EDCs—those able to be analysed in a valid way either in plasma/serum or in urine, thereby enabling evaluation of the health effects of exposure in epidemiological studies.

Please see Table 1 for the groups of EDCs mentioned in this review.

**Table 1:** Overview of the groups of EDCs included in this review

Group	Physical and chemical properties	Example	Common route of exposure	Use in products	Production/regulatory status	Toxicity/mechanism of action
PCBs	209 congeners distinguished by number and position of chlorine atoms substituted on the biphenyl moiety Resistant to acids, bases and heat Most are lipophilic and very persistent	3,3′,4,4′,5-Pentachlorobiphenyl (PCB-126)	High-fat food (dairy, meat, fish)	Mixtures have been used in electrical equipment, surface coatings, inks, adhesives, flame retardants and paints	PCB production was banned by the US Congress in 1979 and by the Stockholm Convention on Persistent Organic Pollutants in 2001 Due to their persistence in the environment, there are still concerns	The chlorination pattern determines the toxicity. Mechanisms of action depend on chlorine substitution pattern of congener: oestrogenic, anti-oestrogenic, neurotoxic, dioxin-like Dioxin-like PCBs are ligands to the AH receptor, while many non-dioxin-like PCBs bind to PXR and CAR
Organochlorine pesticides	Insecticidal properties Highly lipophilic Many are very persistent	DDT and its main metabolite *p*,*p*′-DDE Hexachlorobenzene Several chlordanes	High-fat food (dairy, meat, fish)	DDT was first used during World War II to control lice-borne typhus Subsequently, farmers used DDT to control agricultural pests	DDT was banned in the USA in 1973 and also in some other countries but is still in use in countries with malaria	Wide range of toxic effects *p*,*p*′-DDE, the most environmentally relevant DDT derivative, binds to androgen receptor and has anti-androgenic properties
Dioxins	A diverse range of chemical compounds 419 dioxins and related compounds have been identified Lipophilic Some are very persistent	TCDD	Soil, dairy, meat, seafood	Not used in products Formed during the combustion of wastes or are undesirable byproducts in the manufacture of herbicides, disinfectants and other agents	Covered by the Stockholm Convention on Persistent Organic Pollutants	Wide range of toxic effects, including immune toxicity, developmental and neurodevelopmental effects and changes in thyroid and steroid hormones and reproductive function Only about 30 dioxins are considered to have significant toxicity, with TCDD being the most toxic Mechanisms of action and toxicity vary depending on the chlorine substitution pattern of the congener: oestrogenic, anti-oestrogenic, neurotoxic, dioxin-like The dioxin-like effect is mediated by activation of the AH receptor
BFRs	Widely varying chemical properties At high temperatures, BFRs have an inhibitory effect on combustion chemistry Some are lipophilic and very persistent	Main classes are PBDEs, HBCDDs, TBBPA and other phenols, and PBBs	High-fat food (dairy, meat, fish)	Used in plastics and textile applications, electronics, clothes and insulation in buildings and furniture	The use of certain BFRs is banned or restricted in the EU In the USA, the manufacture of PBB was banned in 1976 Due to the persistence of BFRs in the environment, there are still concerns	Toxic effects, including teratogenicity, carcinogenicity and neurotoxicity, have been observed for some BFR congeners (especially PBDEs) There is evidence that some BFRs disrupt the thyroid hormone system–most data are available for the PBDE class
PFASs	Hydrophobic and lipophobic Some are resistant to environmental degradation and are extremely persistent	Perfluorocarboxylic acids (e.g. PFOA, sometimes called C8, and PFNA) and perfluorosulfonates (e.g. PFOS and PFHxS)	Seafood, drinking water and food contact material	Used in industry and consumer products since the 1950s Used in food packaging materials, non-stick cookware, water-resistant clothing, cleaning products, paints, varnishes and sealants, firefighting foam and cosmetics	Use of PFASs has been largely phased out of food packaging materials The European Parliament has approved an EU directive (2006/122/EU) with restrictions on marketing and use of PFOS and PFOS-related substances	There is evidence that some PFASs disrupt the thyroid hormone system. Some PFASs bind to PPAR-α and to a lesser degree to PPAR-γ.
Bisphenols	Group of non-persistent chemicals with two phenolic rings joined together by a bridging carbon or other chemical structure.	Bisphenol A (BPA; 4,4′-isopropylidenediphenol)	Ubiquitous	Commonly used to produce plastics BPA is used mainly in the manufacture of polycarbonate and is also used in other plastics as a hardener Used in products such as DVDs, dental materials and lunch boxes Epoxy plastic can be used in electronics, building materials, in the protective lining in cans and in the relining of water pipes BPA is present in thermal paper	Controversial issue BPA is banned in baby bottles throughout the EU In 2017, 5.4 million tons of BPA was produced	Initially BPA was designed as a synthetic oestrogen and has been shown to bind to oestrogen receptors (ERα, ERβ, and to the membrane ER) Emerging data shows that BPA interacts with other hormone receptors, including androgen receptors and the thyroid hormone receptor
Phthalates	Esters of phthalic acid Not persistent	DEHP	Ubiquitous	Used as plasticisers in the production of plastics Used in cosmetics, perfumes, pharmaceutical tablets, medical tubing, nutritional supplements, adhesives, paints, food containers and wrappers, toys and cleaning materials	Controversial issue Some countries have banned their use in children’s toys Five million tons of phthalates are produced annually	MEHP, a metabolite of DEHP, has been found in exposed organisms and interacts with all three PPARs

These EDCs were selected because they can be analysed in a valid way either in plasma/serum or in the urine, thereby enabling the evaluation of the health effects of exposure in epidemiological studies

Congeners: congeners are related chemical substances, related to each other by origin, structure, or function

AH, aryl hydrocarbon; CAR, constitutive androstane receptor; EU, European Union; HBCDD, hexabromocyclodecane; MEHP, mono-ethyl-hexyl-phthalate; PBB, polybrominated bisphenol; PBDE, polybrominated diphenyl ether; PFHxS, perfluorohexasulfonate; PFOA, perfluorooctanoic acid; PXR, pregnane X receptor; TBBPA, tetrabromobisphenol A

## Epidemiological studies

Some studies have shown that high POP levels are associated with diabetes; the study that induced a major interest in this topic was conducted by D.-H. Lee and D. R. Jacobs Jr. and colleagues in 2006 [2]. In that cross-sectional study, they used the USA National Health and Nutrition Examination Survey (NHANES) database to show that six different POPs, including PCBs, dioxins and organochlorine pesticides, were related to prevalent diabetes.

Following that landmark study, >40 cross-sectional studies have been published, showing a link between EDC levels and prevalent diabetes in different countries. However, since cross-sectional studies are clearly prone to reverse causation and other biases, prospective studies are warranted.

**Fig. 1:**
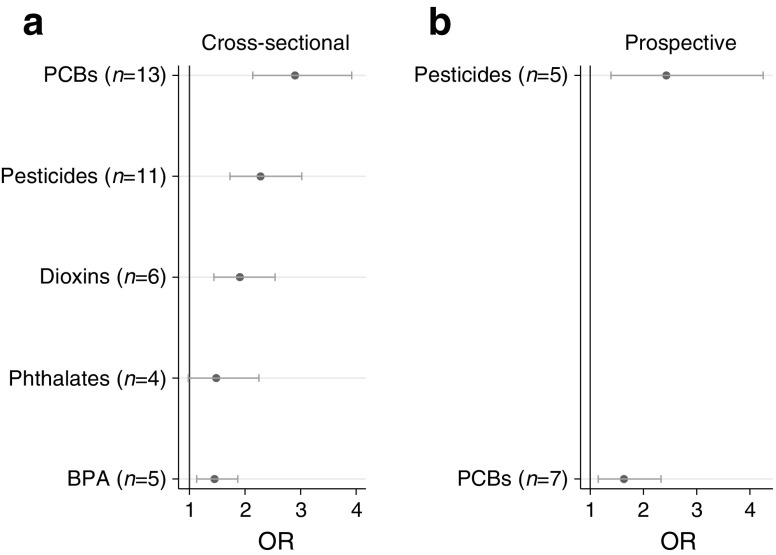
Summary of a meta-analysis of available cross-sectional (**a**) and prospective (**b**) studies on the association between environmental contaminants and diabetes published in 2016 by Song et al [3]. ORs (circles) and 95% CI (horizontal bars) are shown and are based on comparisons between the highest and lowest values presented in the different studies underlying the meta-analysis. The number of studies concerning each of the different chemical classes is shown in parentheses. This figure is available as a downloadable slide

A meta-analysis of the cross-sectional and prospective studies, published in 2016, showed that in the cross-sectional setting significant relationships were found between levels of dioxins, PCBs, organochloride pesticides and BPA and prevalent diabetes, while the relationship for phthalates was of borderline significance [3]. The results of this meta-analysis are summarised in Fig. 1. For the seven prospective studies included in the meta-analysis, data were only available for PCBs and organochlorine pesticides but, as seen in the cross-sectional studies, increased levels of these two classes of contaminants were significantly related to incident diabetes.

In a 2013 US National Toxicology Program workshop review of six studies (including two prospective studies) investigating associations between levels of BFRs and diabetes, no clear association was found [4]. In a review of four cross-sectional studies, no associations were seen between the two most commonly investigated PFASs, perfluorooctane sulfonic acid (PFOS) and perfluorooctanoic acid, and prevalent diabetes, although in the only study investigating perfluorononanoic acid (PFNA) a significant association was found [5]. This relationship between PFNA and diabetes was confirmed in a later cross-sectional study [6]. However, in two prospective studies, PFNA and other PFASs were observed to have no effect or even a protective effect [7, 8].

Taken together, prospective evidence exists for associations between background exposures to PCBs and organochlorine pesticides and incident diabetes. In addition, cross-sectional evidence exists for relationships between dioxins and BPA and prevalent diabetes, while there is no convincing evidence for relationships between phthalates, BFRs or PFASs and diabetes.

## Experimental studies

Several studies indicate that Vietnam veterans exposed to Agent Orange contaminated with dioxins have an increased risk of incident diabetes (see [4] for review) but animal studies have generally shown a hypoglycaemic response to the potent dioxin 2,3,7,8-tetrachlorodibenzo-*p*-dioxin (TCDD) [9]. Neither could any consistent hyperglycaemic responses be seen in rodent studies exploring the effect of PCB exposure [10], except in one study in which dioxin-like PCBs impaired glucose tolerance in lean but not fat mice [11].

Regarding organochlorine pesticides, perinatal exposure to 1,1,1-trichloro-2,2-bis (*p*-chlorophenyl)ethane (DDT) induced impaired glucose tolerance later in life in mice [12]. In addition, adult rodents exposed to DDT later in life showed impaired glucose tolerance and reduced insulin secretion [13].

Nadal and co-workers showed that mice exposed in utero to BPA displayed impaired glucose tolerance and reduced insulin secretion later in life [14]. In addition, long-term exposure of BPA later in life induced impaired glucose tolerance in mice [15].

Taken together, in vivo animal studies do not support the epidemiological evidence of a diabetogenic response to dioxins or PCBs, while evidence in rodents supports an effect for DDT and BPA.

## Potential obesogenic role of contaminants

Based on the experimental finding that the organotin substance tributyltin induced weight gain in mice and that this effect was mediated by peroxisome proliferator-activated receptor (PPAR)-γ and involved reprogramming of mesenchymal stem cells towards the adipocyte lineage [16], it has been proposed that certain environmental contaminants could contribute to the obesity epidemic seen worldwide, acting as so-called ‘obesogens’. Since obesity is the major risk factor for future type 2 diabetes, it is of interest to investigate whether the contaminants found to be related to diabetes might also induce obesity.

The lipophilic POPs, such as dioxins, PCBs and organochlorine pesticides, accumulate in adipose tissue to a major degree. The degree of fat mass influences their circulating levels, making it difficult to study the relationships between these compounds and obesity in adults. However, mother–child cohorts, with measurements in the pregnant mothers and follow-up of the weight gain of the children, may provide evidence for the ‘obesogen’ hypothesis, since it has been suggested that exposure to contaminants early in life would have the greatest impact on future weight gain.

A recent meta-analysis of seven mother–child cohorts showed a positive relationship between prenatal exposure to DDT/dichlorodiphenyldichloroethylene (*p*,*p*′-DDE) and future weight gain in the children [17]. These data are supported by an experimental study showing that prenatal exposure of mice to this pesticide induced reduced energy expenditure and a transient weight gain [12]. When given a high-fat diet, the mice developed impaired glucose tolerance.

In a review from 2011, no convincing association was found between exposure to other types of lipophilic POP and future weight gain in mother–child cohorts [18].

In a recent review of more short-lived, less lipophilic EDCs, prenatal exposure to phthalates and BPA, as well as childhood exposure to these plastic-associated chemicals, was not consistently associated with increased future body weight. In contrast, most studies of PFASs in this context have shown a relationship between prenatal exposure to PFASs and impaired weight gain [19].

Taken together, the best evidence for EDCs inducing obesity that might cause diabetes is present for the pesticide DDT.

## Mechanistic studies

Insulin secretion and insulin sensitivity are the two main characteristics determining glucose tolerance. In a recent study, the early and late insulin response was modelled using data obtained at a 2 h OGTT [20]. Both the dynamic first-phase and the static second-phase insulin response were impaired in non-diabetic individuals with high levels of organochlorine pesticides, while the association with PCBs was weaker. When the proinsulin-to-insulin ratio was used as a proxy for a disturbed beta cell function, high levels of some phthalates and some PFASs were associated with this ratio [6, 21].

In experimental studies, the potent dioxin TCDD has been shown to influence insulin secretion in a rat insulin-secreting beta cell line and in isolated rat pancreatic cells [22]. TCDD has also been reported to induce pancreatic cell death by autophagy in the beta cell line INS-1E [23]. In insulin-sensitive tissues such as liver, skeletal muscle and adipose tissue from the guinea pig, TCDD induced insulin resistance [24], possibly by downregulation of glucose transporters [25]. The insecticides malathion and diazinon have been reported to influence insulin-secreting beta cells [26, 27], partly by downregulation of muscarinic receptors. Prenatal exposure of rats to di(2-ethylhexyl) phthalate (DEHP) has been shown to induce hyperglycaemia, hyperinsulinaemia and a reduced beta cell mass [28]. In vitro, organochloride pesticides reduced insulin secretion in pancreatic INS-1E beta cells [20].

Using the HOMA index of insulin sensitivity in humans, a prospective study showed insulin sensitivity to be impaired in middle-aged individuals who 20 years earlier showed high levels of organochlorine pesticides [29]. Other cross-sectional studies have linked levels of PCBs and, especially, organochlorine pesticides to insulin sensitivity [20]. Although some phthalates and PFASs have been linked to impaired insulin sensitivity in humans, some studies have failed to reproduce the link between PFASs and insulin resistance [6, 7, 21, 30].

Experimental prenatal exposure to PFOS, as well as to DDT, phthalates and BPA, resulted in impaired insulin sensitivity and glucose intolerance [12, 14, 31]. In addition, exposure to a mixture of PCBs, or a mixture of 23 lipid-soluble POPs, induced glucose disturbances and insulin resistance in vivo in mice [32, 33]. The effect of a mixture of POPs has also be observed in vitro in cultured adipocytes [33].

Taken together, many of the EDCs investigated have been shown to impair insulin secretion and sensitivity in both human and experimental studies.

## Discussion and future perspectives

Ideally, large prospective studies are needed to investigate whether fetal and prenatal exposure to EDCs induces obesity and later type 2 diabetes. Unfortunately, those studies are not likely to be conducted for practical reasons. Since an ideal study would take 60–70 years to accomplish, most of the chemicals used at the initiation of the study would probably not be in use at the time when the results became available. Thus, in practice, we do have to rely on results from studies not covering the whole lifespan.

Currently, results of a moderate number of prospective studies involving PCBs and organochlorine pesticides are available. It is reassuring that the results of a meta-analysis of these studies are in line with results from a much larger number of cross-sectional studies on the same EDCs. Firm experimental data also exist to support a causal relationship between DDT/*p*,*p*′-DDE exposure and diabetes development. According to the well-established Grading of Recommendations Assessment, Development and Evaluation (GRADE) criteria used for reviewing evidence (http://www.gradeworkinggroup.org), moderate evidence exists of a true relationship between DDT/*p*,*p*′-DDE exposure and diabetes development. To achieve a high degree of evidence, randomised trials are needed but will never be accomplished in the field of EDC research. Regarding dioxins and PCBs, it is likely that the rodent models used are not appropriate due to the large discrepancy in sensitivity of the aryl hydrocarbon receptor between humans and rodents and therefore there is less evidence than for DDT/*p*,*p*′-DDE. For other EDCs, the evidence is low, since we do not have enough prospective data.

A very important future aim is to gain prospective data in population-based studies with a high number of incident outcomes. The major hurdle in achieving that goal is not the lack of such cohorts but rather that the volume of blood/urine needed for analysis of EDCs is still too large. Only when it is possible to measure EDCs in <100 μl of plasma at a reasonable cost will biobanked plasma from large cohorts be used for this purpose. Although analytical chemistry has improved over the years regarding the volumes needed for proper analyses, the advancement of the field is heavily dependent on further developments.

Another area in need of improvement is the study of mixture effects. Nowadays, the relationships between EDCs and diseases, such as diabetes, are usually studied either by the sum of concentration of the chemicals in a certain class or by simply analysing the contaminants one by one. One exception to this rule is the use of toxic equivalents, based on binding to the aryl hydrocarbon receptor, but this ‘pharmacological approach’ is only applicable to dioxins and other dioxin-like chemicals. In other instances, sophisticated statistical methods are needed to study mixture effects and some examples of using those methods to study mixture effects have been published [34, 35]. It is also possible to use less sophisticated methods to estimate the effects of multiple EDCs once they have been identified one by one, as exemplified by our recent estimation in the Prospective Investigation of the Vasculature in Uppsala Seniors (PIVUS) study that the population attributable risk for diabetes for the combination of 2,2′,4,4′,5,5′-hexachlorobiphenyl (PCB-153), *p*,*p*′-DDE, monoethylphthalate and PFNA was 13%. The impact of these contaminants was estimated to be related to a yearly healthcare cost of diabetes in Europe of €4.51 billion [36]. This is in line with the findings of an expert panel, based on several published studies, who concluded that the cost of obesity and diabetes caused by EDCs was €18 billion a year [37].

Since it is impossible to obtain the highest degree of evidence by performing randomised clinical trials with environmental contaminants, it is essential to support epidemiological findings with animal data to establish causality. Therefore, use of relevant models using animals having similar sensitivity to the different chemicals as humans is critical to increase the level of confidence in human findings.

At first glance it seems contradictory that aryl hydrocarbon receptor activation (dioxins), androgen receptor antagonism (*p*,*p*′-DDE) and PPAR-γ binding (phthalates and PFASs) all could lead to disturbed glucose metabolism but it must be remembered that a number of pharmaceutical drugs with different modes of action can also impair glucose tolerance (e.g. β-blockers, thiazide diuretics and neuroleptic drugs). Several downstream effects of the agonistic/antagonistic actions on the receptors described above, such as mitochondrial dysfunction [38], inflammation [39], oxidative stress [40] and alterations in thyroid and cortisol pathways [41], comprise possible mediating pathways linking different EDCs to glucose disturbances.

In conclusion, several epidemiological studies have pointed to an association between EDCs and diabetes. According to the principle of REACH, a European Union regulation of chemicals (no. 1907/2006) designed to ensure a high level of protection of human health and the environment, this might be enough for regulatory authorities to regard this problem as serious. The best evidence for an association between EDCs and diabetes, graded as moderate, is found for DDT/*p*,*p′*-DDE. A lower grade of evidence is found for PCBs, since supporting experimental data are lacking. For other EDCs, prospective studies are needed to support the findings of existing cross-sectional studies.

### Contribution statement

Both authors were responsible for drafting the article and revising it critically for important intellectual content. Both authors approved the version to be published.

## Electronic supplementary material

ESM (PPTX 78.5 kb)

## Abbreviations

BFR: Brominated flame retardant
BPA: Bisphenol A
DDT: 1,1,1-Trichloro-2,2-bis (*p*-chlorophenyl)ethane
DEHP: Di(2-ethylhexyl) phthalate
EDC: Endocrine-disrupting chemical
GRADE: Grading of Recommendations Assessment, Development and Evaluation
*p*,*p*′-DDE: Dichlorodiphenyldichloroethylene
PCB: Polychlorinated biphenyl
PFAS: Perfluoroalkyl and polyfluoroalkyl substance
PFNA: Perfluorononanoic acid
PFOS: Perfluorooctane sulfonic acid
POP: Persistent organic pollutant
PPAR: Peroxisome proliferator-activated receptor
TCDD: 2,3,7,8-Tetrachlorodibenzo-*p*-dioxin
