# Supplementary material for: Endocrine-disrupting chemicals and risk of diabetes: an evidence-based review
Source: Diabetologia. 2018 May 9;61(7):1495–502. doi: 10.1007/s00125-018-4621-3 (PMC6445457; doi:10.1007/s00125-018-4621-3)
Supplement: Supplementary file 1 — (PPTX 78.5 kb) [file 125_2018_4621_MOESM1_ESM.pptx]

## Slide 1
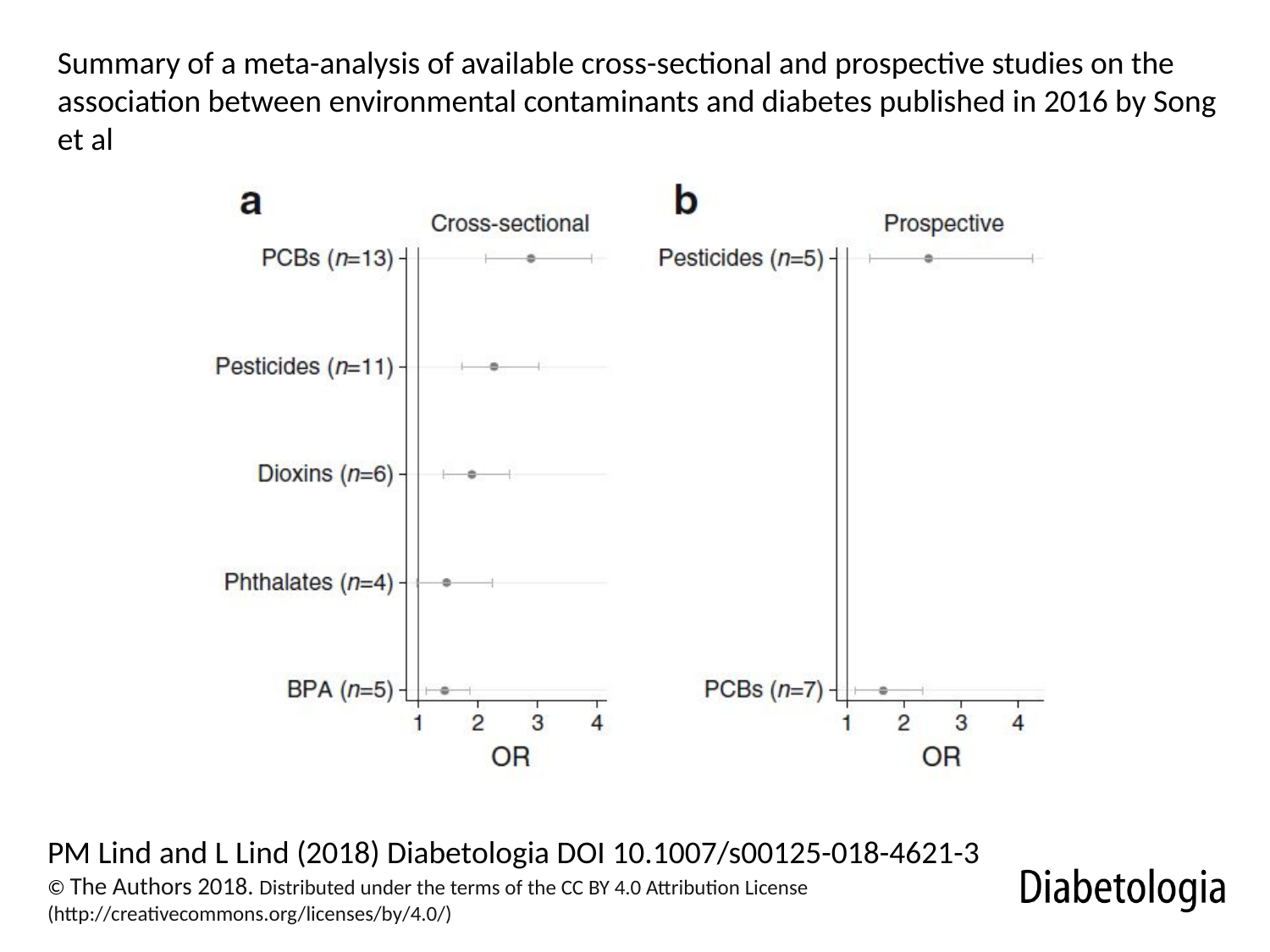

Summary of a meta-analysis of available cross-sectional and prospective studies on the association between environmental contaminants and diabetes published in 2016 by Song et al
PM Lind and L Lind (2018) Diabetologia DOI 10.1007/s00125-018-4621-3
© The Authors 2018. Distributed under the terms of the CC BY 4.0 Attribution License (http://creativecommons.org/licenses/by/4.0/)
